# A Model for Integrating Elementary Neural Functions into Delayed-Response Behavior

**DOI:** 10.1371/journal.pcbi.0020025

**Published:** 2006-04-07

**Authors:** Thomas Gisiger, Michel Kerszberg

**Affiliations:** 1 Récepteurs et Cognition, Institut Pasteur, Paris, France; 2 Modélisation dynamique des systèmes intégrés, CNRS UMR 7138, Université Pierre et Marie Curie, Paris, France; University College London, United Kingdom

## Abstract

It is well established that various cortical regions can implement a wide array of neural processes, yet the mechanisms which integrate these processes into behavior-producing, brain-scale activity remain elusive. We propose that an important role in this respect might be played by executive structures controlling the traffic of information between the cortical regions involved. To illustrate this hypothesis, we present a neural network model comprising a set of interconnected structures harboring stimulus-related activity (visual representation, working memory, and planning), and a group of executive units with task-related activity patterns that manage the information flowing between them. The resulting dynamics allows the network to perform the dual task of either retaining an image during a delay (delayed-matching to sample task), or recalling from this image another one that has been associated with it during training (delayed-pair association task). The model reproduces behavioral and electrophysiological data gathered on the inferior temporal and prefrontal cortices of primates performing these same tasks. It also makes predictions on how neural activity coding for the recall of the image associated with the sample emerges and becomes prospective during the training phase. The network dynamics proves to be very stable against perturbations, and it exhibits signs of scale-invariant organization and cooperativity. The present network represents a possible neural implementation for active, top-down, prospective memory retrieval in primates. The model suggests that brain activity leading to performance of cognitive tasks might be organized in modular fashion, simple neural functions becoming integrated into more complex behavior by executive structures harbored in prefrontal cortex and/or basal ganglia.

## Synopsis

Before we do anything, our brain must construct neural representations of the operations required. Imaging and recording techniques are indeed providing ever more detailed insight into how different regions of the brain contribute to behavior. However, it has remained elusive exactly how these various regions then come to cooperate with each other, thus organizing the brain-scale activity patterns needed for even the simplest planned tasks. In the present work, the authors propose a neural network model built around the hypothesis of a modular organization of brain activity, where relatively autonomous basic neural functions useful at a given moment are recruited and integrated into actual behavior. At the heart of the model are regulating structures that restrain information from flowing freely between the different cortical areas involved, releasing it instead in a controlled fashion able to produce the appropriate response. The dynamics of the network, simulated on a computer, enables it to pass simple cognitive tests while reproducing data gathered on primates carrying out these same tasks. This suggests that the model might constitute an appropriate framework for studying the neural basis of more general behavior.

## Introduction

An important unanswered question in neurobiology is how neural activity organizes itself to produce coherent behavior. Lesion, electrophysiological, and imaging studies targeting specific cognitive functions have provided very detailed insights into how different regions of the brain contribute to behavior. More specifically, they have shown the role of various regions of cortex in implementing functions such as visual representation of stimuli [1,2], sustainment of the memory of a stimulus [3], representation of tasks [4] or abstract rules [5,6], selection of a response among a set of possibilities [7,8], shielding of memory from distractions [9], and planning of movements [10]. However, it remains unclear how the different regions of cortex interact together to build even the simplest behavior. Indeed, even the elementary action of looking at an object and preparing to reach for it requires a cascade of neural processes that have to take place in the right order and with the proper timing to be successful.

Here, we propose that adequate behavior can be generated from the set of functions mentioned above if the information these cortical regions contain and exchange with each other is managed by executive or control structures in a manner suiting the task at hand. Brain-scale activity coding for integrated behavior might then be constructed by these executive units, from a repertoire of simple neurocognitive functions, which would be selected, recruited, ordered, and synchronized to implement the necessary neural computations.

**Figure 1:**
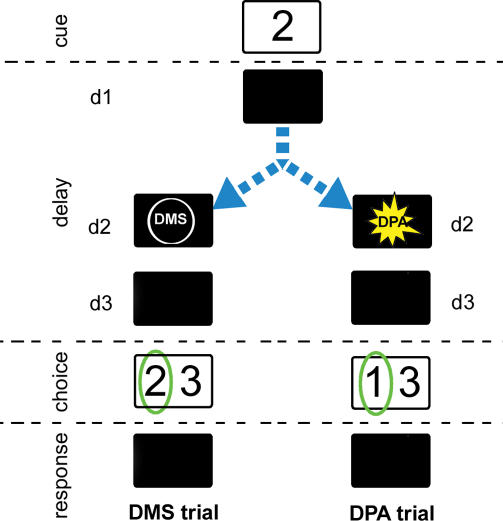
Diagram of the MDR Task The MDR task consists of randomly mixed DMS and DPA trials [11]. The stimuli used in the simulations are represented by numbers 1, 2, 3, and 4. A sample image is presented during the cue period and then hidden during the delay. A task signal specifying which task the subject is expected to perform is briefly displayed during the delay (subdelay d2). Two images are then presented during the choice period: a target (circled in green) and a distractor. The target image depends on the trial type: it is identical to the sample image in DMS trials, and it is the sample's paired-associate image in DPA trials. In DPA trials, images have been associated in the arbitrarily chosen pairs {1,2} and {3,4}. If the subject chooses the target image, it will receive positive reward during the subsequent response period. Otherwise, negative reward is dispensed to the subject. Note that the task signal is not essential to trial success: the subject can figure out after the delay which task it is required to perform by inspecting the proposed stimuli. The signal, however, gives the subject the opportunity to act prospectively and to anticipate the target during the delay. Length of trial periods used: cue, 0.5 s; delay (divided in subdelays: d1, 0.3 s; d2, 0.4 s; and d3, 1.0 s); choice, 0.5 s; and response, 0.5 s.

**Figure 2:**
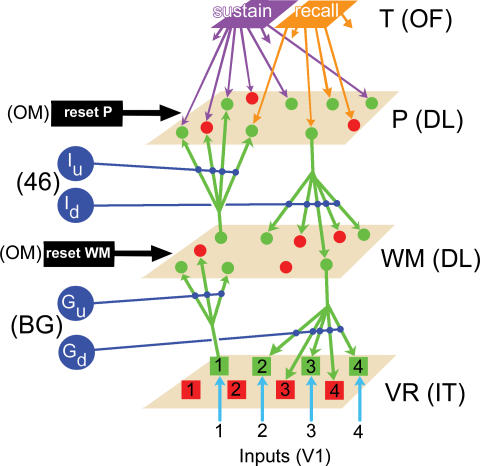
Diagram of the Structure of the Network Excitatory and inhibitory neurons (represented by green and red dots, respectively) are arranged in two-dimensional layers P and WM and interconnected by short-distance connections (not shown). Layer VR is composed of four excitatory units (green squares), each representing a group of cortical cells coding for a single image, and four inhibitory units (red squares), which implement lateral inhibition on excitatory VR units. Layers P, WM, and VR are connected via diffuse and homogenously distributed vertical excitatory projections (green arrows). All connections in the network are fitted with standard Hebbian learning algorithm, while downward connections have in addition reinforcement learning. Each P neuron receives a single priming connection from either the sustain (represented by a violet arrow) or recall (orange arrow) unit. Layers WM and P are the targets of reset units (reset WM and reset P) which, when active, reinitialize to zero the membrane potential and output of all neurons in the layer. Units G_u_, G_d_, I_u_, and I_d_ gate activity which travels from VR to WM, WM to VR, WM to P, and P to WM, respectively (dark blue lines). This gives the network the freedom to either transfer information from one layer to another, or to isolate layers so that they can work separately. Visual information from the exterior world enters the network via the Inputs variables, which feed stimulus-specific activity into layer VR (turquoise arrows). Letters between parentheses indicate tentative assignation of network components to cortical or subcortical areas (see Discussion). 46, area 46; BG, basal ganglia; DL, dorsolateral; IT, inferotemporal; OF, orbitofrontal; OM, orbitomedial; V1, primary visual cortex. Network areas and layers: P, planning; T, task; VR, visual representation; WM, working memory.

**Figure 3:**
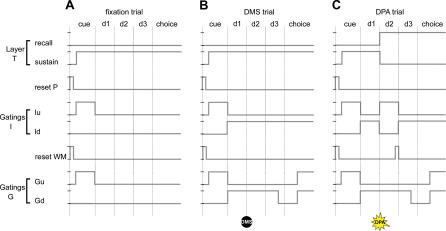
Dynamics of Task, Reset, and Gating Units The complex overall activity of the network is directed by the coordinated binary firing (either “on” or “off”) of control units, each implementing a basic function: gatings regulate the upward and downward flow of information, resets bring processors back to a null state of activity, and the task units prime activity in layer P. The units' firing patterns are grouped in three sets, each corresponding to a different task: one for fixation trials (A), one for DMS trials (B), and one for DPA trials (C). These firing patterns specify the neural computations performed by the network to pass each task. Note that task parameters and notations are as in Figure 1, and that the activity of each control unit during the response period (which is not shown here for clarity) is identical to that during the choice period. Control unit notations are as in Figure 2. (A) The fixation task only requires that the network observes the sample image presented at the beginning of each trial. To do this, the network first clears from its WM and P layers any activity left over from the preceding trial, and then allows visual information from the presented sample to rise into these layers. (B) The DMS task generalizes the fixation task, requiring that the network retains the observed sample image during a delay to then match it against target and distractor images during the choice period. These operations are implemented by the above additional activities in the firing patterns of gatings I_d_, G_u_, and G_d_ (see “Analysis of MDR Task Performance” for details). (C) The DPA task is identical to DMS except that the network needs to retrieve during subdelays d2 and d3 the image associated with the sample. This recall process is implemented during these periods by additional activities for gatings I_u_ and I_d_, and the reset WM unit (see “Analysis of MDR Task Performance” for details).

To illustrate this hypothesis, we present a neural network model able to pass the mixed-delayed response (MDR) task, which was introduced to study memory retrieval in the monkey using visual associations [2,11,12]. This task (Figure 1) consists of randomly mixed delayed-matching to sample (DMS) and delayed-pair association (DPA) trials [2,12], which require that the subject either maintain the memory of an image during a delay, or remember an image associated with it, respectively. Which type of trial is to be performed is signaled to the subject during the delay. The network contains structures harboring image-related activities, each of them implementing one of the elementary functions crucial for task execution: visual representation, working memory, and planning memory (Figure 2). These areas are complemented by executive units, which control the activity held in these layers and regulate the information flow between them. The neural computations necessary to perform the tasks are encoded in the firing patterns of these control units, which are coordinated and depend strongly both on the trial type and the phase of the trial (Figure 3). The resulting dynamics allows the network to be trained for the DMS and then for the DPA task while reproducing electrophysiological data gathered on the inferior temporal (IT) [2,11] and prefrontal (PF) [12] cortices of monkeys performing similar tasks.

## Results

### Model Description

The model (see Figure 2) has several components in common with an earlier, simpler network used to reproduce DMS execution [13]: these elements are a working memory (WM), a visual representation area (VR), and an input layer (Input), which simulate parts of the PF, IT, and primary visual cortices, respectively. WM is a two-dimensional layer of excitatory and inhibitory neurons interconnected by short-range projections. It implements in the model a memory buffer able to retain an image using stable neural activity sustained through feedback excitatory connections. Layer VR contains stimulus-specific neurons that represent the visual components of the images used in the task, either when they are perceived or retained by the network. It receives top-down connections from layer WM, and bottom-up connections from the lower visual area layer Input. It is through Input that “visual” information enters the network. The flow of information in the network is managed by signal gating and memory reset circuits: gating G_u_ controls visual information traffic along upward connections going from VR to WM. Similarly, G_d_ gates memory signals traveling downward from WM to VR. The “reset WM” unit, when active, suppresses all ongoing activity in layer WM. We have shown earlier that when the G_u_, G_d_, and reset WM units obey suitable firing patterns (Figure 3B, gatings G and reset WM), the resulting network can be trained for the DMS task using simple Hebbian [14] and reinforcement-learning algorithms, which it then passes with a high degree of success [13].

The present model expands on this circuitry, placing it partly under the control of hierarchically higher systems: a task layer (T), a planning memory layer (P) together with its own reset module (reset P), and two new gatings, I_u_ and I_d_ (Figure 2).

The new task layer T consists of two units with mutually exclusive activities, sustain and recall. It forms the neural implementation of what we propose is the absolute minimal set of processing actions necessary to pass the DMS and DPA tasks. The sustain unit is active whenever the network maintains a memory of the sample image. It is therefore active throughout DMS trials since in this case the sample image is also the target, and during the first half of DPA trials. By contrast, the recall unit is active only when the network recalls the image associated with the sample and maintains its representation in working memory. It is therefore always silent except during the second half of DPA trials, where the network performs target retrieval (Figure 3B and C, layer T).

The planning layer P is a higher level working memory with circuitry identical to that of WM, including its own reset unit. P neurons receive excitatory connections from layer WM (Figure 2). In addition, each P cell receives a “priming” connection from one of the task units. We define a priming connection as a projection that keeps the target cell from firing if the priming unit is silent. Therefore, if a P cell is depolarized above its firing threshold, that cell can fire only if the task unit that primes it is also active. Which of the sustain and recall units primes a given P neuron is chosen at the start of the simulation by a random process using equal probabilities for both units. Thus, P neurons are divided into two sets of cells diffusely distributed all over the layer: one set primed by the sustain unit and the other by the recall unit. P cells therefore act as coincidence detectors: their firing is determined both by which sample image is held in working memory (through the projections from WM) and whether the network is currently engaged in sample sustaining or target retrieval (via the priming connections from T). This firing is transmitted back to the working memory by descending connections onto layer WM. As illustrated when describing network dynamics, the overall connectivity between layers T, P, and WM makes activity in layer P the neural correlate of the “planning” or of the “project,” formed by the network, to use a given image as target for the trial.

The flow of information between layers WM and P is regulated by gating areas I_u_ and I_d_ (Figure 2). When open, I_u_ allows memory signals in layer WM to rise to P and elicit new planning activity there. Conversely, when active, I_d_ permits planning signals to travel down to working memory, where it potentiates the representation of the image regarded as the upcoming target. These gatings are complemented by the reset P unit which, when firing, eliminates any activity in layer P (Figure 2, gatings I and reset P).

The WM–VR circuitry enables the network to perform the operations of sample recognition, sample storage, and the selection as target of the image currently held in working memory. These circuits are now complemented by the novel layers P and T, which allow autonomous modification by the network itself of the content of working memory. The necessary neural computations are guided by the ensemble of gating and reset units and their firing patterns (Figure 3). These activity patterns, which are defined at the start of simulations, allow the network, after preliminary fixation trials (see below and Figure 3A), to be trained for the DMS, DPA, and MDR tasks, which it then passes with a high degree of success.

### Learning Phase

Initially, with all connections of equal strength, the model performs the tasks at no better than chance level, even though the control units already possess their mature firing patterns (Figure 3). The learning procedure consists in three consecutive stages: training for fixation, DMS, and DPA tasks.

The fixation task (Figure 3A) is identical to the DMS task, except that no task signal or images are presented during the delay and choice periods, respectively, and accordingly no reward is dispensed: the network is only required to “observe” the sample image presented during the cue period of each trial (drawn at random from the set of four images 1, 2, 3, and 4; see Figure 1). The model needs this preliminary training to prepare its working and planning memories for the upcoming DMS and DPA tasks. Indeed, during fixation training, Hebbian synaptic plasticity leads to the emergence in layer WM of four neural circuits or clusters (one for each image used in the task) with self-sustainable and stimulus-specific activity. The same process also produces four neural clusters among the subset of P neurons primed by the sustain unit of layer T. Each of these circuits develops a center-surround structure (i.e., exhibiting a core of interconnected excitatory neurons able to sustain their activities) surrounded by an inhibitory region acting as lateral inhibition within the layer [15]. Only a few trials, about 20 or so, are necessary for these emerging structures to become stable enough to withstand without collapsing the upcoming neural computations necessary to pass the DMS and DPA tasks.

The network is then subjected to DMS training. There, the neural assemblies mentioned above, which act as building blocks for the network, are connected (together and with units of layer VR) to form macrocircuits spanning the whole system: the clusters present in layer WM now form neural representations of the images used for the task, while each circuit in layer P codes for the plan to use a different sample image as target. This latter phase occurs as a result of the reinforcement-learning algorithm that modulates the vertical connections and makes use of the positive and negative reward signals dispensed to the network at the end of each trial to reinforce the productive connections. Connections whose operation tends to increase positive reward become stronger and therefore end up dominating the dynamics of the model. Successful DMS training takes an average of about 41 trials (SD = 19), producing a network that passes DMS with a success rate of 90% or more.

DPA task learning follows last (Figure 1, DPA trial), during which four additional neural circuits are created in layer P, but this time among the subset of neurons primed by the recall task unit. These new clusters are then integrated in the pre-existing macrocircuits by connecting themselves with the image-specific clusters already present in the working memory layer. As illustrated below, the connections between the new P clusters and the already established WM layer clusters code for the associations of images in pairs. More precisely, clusters of recall-primed P neurons code for the *plans* to use as targets, not the sample images themselves, but the images associated with the samples instead. The associations are learned through a process of trial and error where the network attempts to associate with the sample an image picked at random among those presented after the delay. Positive reinforcement secures the correct associations by strengthening the connections that led to the right choice (which images constitute correct pairs is determined by the experimenter before training: we chose pairs {1,2} and {3,4}; see Figure 1). Each pair is learned as two separate associations. Thus, the association of sample image 1 with target image 2 is coded by a neural circuit distinct from that implementing the association between sample image 2 and target image 1. Successful DPA training takes an average of about 58 trials (SD = 33). The resulting network passes the DPA task, and hence the MDR task, with a success rate of 90% or more.

We found that in approximately 50% of runs, the network successfully learned all tasks. In 4%–6% of runs, the network did not learn any task at all while in the remaining runs the network managed to pass DMS only.

### Analysis of MDR Task Performance

For further details on the network's dynamics, the reader is referred to short movies of the network passing DMS and DPA trials available on the website of the journal (see Videos S1 and S2).

#### DMS trial.

**Figure 4:**
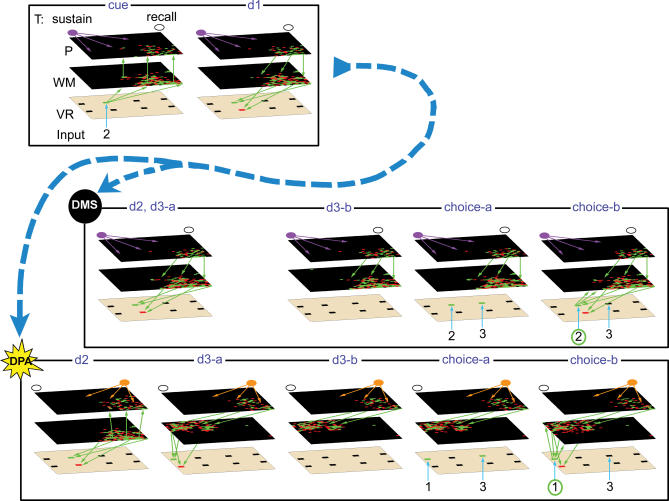
Snapshots of the Network Passing DMS and DPA Trials For easier comparison, the two trials share the same sample (image 2) and distractor (image 3) but differ by their targets (circled in green, DMS trial: 2, DPA trial: 1). The layers T, P, WM, and VR of the network are represented in three dimensions, one on top of the other. Green (red) squares in layers P, WM, and VR represent firing excitatory (inhibitory) neurons. The green arrows represent a sample of the vertical connections strengthened during the learning phase, when the gating controlling them is open. Task units are represented as follows: empty dot represent inactive units; violet and orange dots represent active sustain and recall units, respectively. Priming arrows (violet and orange arrows) are only represented when the corresponding task unit is active. Task parameters and notations are as in Figures 1 and 2. Notations for network areas are as in Figure 2. For clarity, the state of the network during the response period has not been displayed. It is similar to that during the choice period. d3-a, first 0.8 s of subdelay d3; d3-b, last 0.2 s of subdelay d3; choice-a, first 0.2 s of choice period; choice-b, last 0.3 s of choice period.

Each trial starts with the presentation through the Input layer of a sample image, which is stored in the network as neural activity spanning the VR and WM layers (i.e., a representation of this image; see Figure 4, cue—image 2 was chosen as sample image in both trials depicted on Figure 4). Activity then spreads further to the planning layer where it triggers neurons primed by the sustain unit. These firing P neurons, together with the connections they receive from the cluster in layer WM representing image 2 and those they project back onto it, form the neural correlate of the current plan or project by the network to choose sample image 2 as target for the trial.

This plan is set into motion during subdelay d1, as image 2 is hidden (Figure 4, d1). At that time, the network maintains in its VR and WM layers a representation of image 2, which is further reinforced by the excitatory input received from the activity present in the planning layer.

At the beginning of subdelay d2, a signal is presented to the subject to indicate whether the current trial is of type DMS or DPA. In the case of live subjects, this signal is conveyed by a change in the monitor's screen color [11]. In the simulations, we do not model the signal itself or its perception by the network. Modeling starts at the level of the interpretation of the task signal and the subsequent choice of processing actions necessary to pass the trial. In the present case, the choices available to the network were either to continue to sustain the representation of image 2, or to start focusing on its paired-associate, image 1.

In the case of a DMS trial (Figure 4, DMS d2), the target image is identical to the sample presented before the delay. The plan to choose image 2 as the target is still active in layer P and therefore no change in the activity of the network is necessary. The network then merely waits until the end of the delay to select image 2 as the target (Figure 4, DMS d3-a).

At the end of subdelay d3, the influence of working memory layer over the visual representation layer is momentarily blocked (Figure 4, DMS d3-b) to let VR neurons analyze the images presented during the choice period: in the present case, image 2 was the target and image 3 the distractor (Figure 4, choice-a). Control of WM over layer VR is then restored, making the network choose the image corresponding to the representation it currently holds in its working memory (i.e., target image 2; Figure 4, DMS choice-b). This process of target and distractor perception followed by target selection has been described in detail previously [13].

#### DPA trial.

Alternatively, if the task signal indicates a DPA trial, the target will not be image 2, but rather its paired associate, image 1. Since this image has to be retrieved from memory, the network first switches activity in the task layer from the sustain to the recall unit (Figure 3C, d2). This switching starts by eliminating from layer P the ongoing activity of the subset of neurons primed by the sustain unit, which coded for the plan to use image 2 as target (Figure 4, DPA d2). At the same time, information flow is redirected from WM toward layer P. The representation of image 2, which is still present in working memory, triggers in layer P the firing of a different cluster formed of cells primed by the recall unit (Figure 4, DPA d2). This assembly, which therefore receives connections from the WM cluster corresponding to image 2, projects back onto the cluster in WM specific for image 1 (i.e., the image associated with 2 during DPA training). The new planning activity therefore encodes the project of inducing in layer WM the representation of image 1, so as to use image 1 as target for the trial.

Before this is done, however, the network first removes, at the beginning of subdelay d3, the representation of image 2 from working memory. This is necessary to make way for the weaker top-down signal inducing activity specific to image 1. Then, by directing once again the information flow from layer P to layer WM, the planning activity generates in working memory the representation of target image 1 (Figure 4, DPA d3-a). This completes the retrieval (or recall) of image 1 via its visual association with image 2.

The last phase of the trial (i.e., the target perception and selection) proceeds exactly as for the DMS trial above, except that the network now chooses image 1 as target instead of image 2 (Figure 4, DPA d3-b to choice-b).

### Analysis of Neural Activity

#### Layer VR cells.

**Figure 5:**
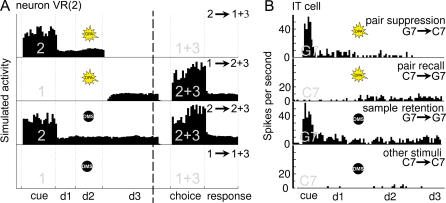
Comparison of the Response from the Model's Excitatory Unit VR(2) with the Observed Activity of a Cortical Neuron Comparison between simulated (A) and experimental (B) units is restricted to the cue and delay periods (left of the dashed line on theoretical data). Activities to the right of the dashed line (choice and response periods) are predictions of the model. Notation: x → y + z = trial with sample x, target y, and distractor z. Other notations are as in Figures 1 and 2. (A) Activity of excitatory unit 2 of visual representation layer VR during DMS and DPA trials where images 1 and 2 are used as sample and target (see Materials and Methods for a definition of the activity of VR units). Task parameters: cue, 0.5 s; d1, 0.3 s; d2, 0.4 s; d3, 1 s; choice, 0.5 s; response, 0.5 s. (B) Recordings of a single IT neuron during four pair-association with color switch [11] trials with images G7 and C7 used as sample and target (sample image written in light gray). Images G7 and C7 form a pair in DPA trials. Pair suppression: DPA trial with sample image G7; pair recall: DPA trial with sample image C7; sample retention: DMS trial with sample image G7; other stimuli: DMS trial with sample image C7. Task parameters: cue, 0.5 s; d1, 2 s; d2, 3 s; d3, 1s. The task signal (black dot or bright star) represents on the figure the signal which indicates to the monkey the current trial type. (Modified from Figure 2 of [11].)

The activity during DMS and DPA trials of excitatory layer VR cells is summarized in Figure 5A. VR inhibitory cells firing is identical to that of excitatory cells except that it does not possess the bottom-up component exhibited by the latter during the sample and first part of the choice periods.

VR cell firing comprises two components. The first component is produced by the one-to-one connections that rise from the Input layer representing lower visual areas (Figure 2). It is bottom-up in nature, being triggered every time the network is presented the corresponding image, whether it is the sample, target, or distractor. The second component is of top-down origin, being produced by the downward connections which WM neurons project onto the VR layer. As exemplified below, this component emerges during DMS and DPA training, making VR neurons act like an extension in visual cortex of the representation of images held in the working memory layer. A given VR cell will therefore fire whenever the image it corresponds to is displayed to the network, or if its representation is currently held in working memory.

Figure 5A shows the firing pattern of excitatory cell VR(2) during typical DMS and DPA trials. For the DMS task, the cell fires continuously throughout trials where image 2 is used as sample and target (Figure 5A, curve 2 → 2 + 3). If, however, the trial features a different image than image 2, then another VR cell will fire and unit VR(2) will therefore remain silent (Figure 5A, curve 1 → 1 + 3).

VR activity during DPA trials is more complex, reflecting the changes in neural activity taking place in layer WM during the recall of the target image. As a result, if image 2 is used as sample (Figure 5A, curve 2 → 1 + 3) VR(2) firing is limited to the cue and subdelays d1 and d2 periods of the trial. This is a consequence of the representation of image 2 which is first evoked (cue period), then sustained (subdelays d1 and d2) and finally eliminated from layer WM (start of subdelay d3) to make way for new firing coding for target image 1 (i.e., the image paired with image 2 during training). VR cells being incapable of autonomously sustaining their firing, the activity of cell VR(2) then collapses.

Conversely, in DPA trials where image 2 is the target and has to be recalled from its association with sample image 1, cell VR(2) is first silent (periods cue and subdelays d1 and d2) but then starts firing at the beginning of subdelay d3 when the representation of image 2 is generated through recall in layer WM (Figure 5A, curve 1 → 2 + 3).

#### Layer WM cells.

**Figure 6:**
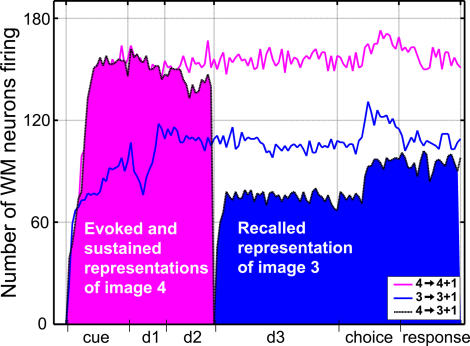
Comparison of Evoked, Sustained, and Recalled Image Representations in Layer WM Each line represents the number of active WM neurons at any instant of a particular trial. The pink and blue curves correspond to DMS trials, where images 4 and 3 are used as sample, respectively. These curves illustrate that different neural assemblies represent the sample image when it is perceived by the network, or when this representation is subsequently sustained. For instance, when image 3 is used as sample (blue curve), fewer cells are mobilized by the presentation of the image (cue period) than by its memory sustained during the delay. The black curve corresponds to the DPA trial where the sample and target are images 4 and 3, respectively. The pink area indicates the amount of WM cells mobilized by the evoked and sustained representations of image 4. The blue area denotes the number of cells making up the representation of image 3 recalled by association. This latter representation, in the case of image 3, clearly mobilizes fewer neurons than either the evoked or sustained representations of that same image.

WM cells exhibit firings that are both image specific and strongly dependent on the trial type and trial period. These characteristics are illustrated at the population level by Figure 6, which displays the number of active WM cells during DMS and DPA trials sharing either the same samples or identical targets.

**Figure 7:**
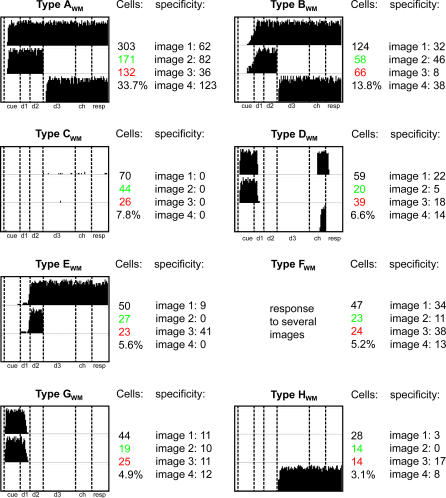
List of the Eight Most Common Neural Responses of WM Layer Neurons Each of the boxes contains three raster plots (top, center, and bottom rows), which represent the response of a single cell to a DMS trial where the cell's preferred image is the sample (top of each box), a DPA trial where the cell's preferred image is the sample (center row in each box), and a DPA trial where the cell's preferred image is the target (bottom). Cells: For each class are listed the total number of cells in the class (black), the number of excitatory/inhibitory cells in the class (green/red), and the percentage of the total number of WM neurons the content of this class represents. Specificity: details the number of cells responding to each of the four images. Note that each type F_WM_ cells responds to more than one image. The cells contained in these eight classes represent a sample of 725 cells out of the 900 contained in layer WM. We found that a comprehensive classification of the cells' activities require at least 27 classes to be complete and that the distribution of cells in these classes follows a power law (see Figure 8 and Discussion).

The strong stimulus specificity of WM cells is demonstrated by the different number of neurons that fire during DMS trials featuring different sample images. For instance, in Figure 6, more cells fire during trial 4 → 4 + 1 than trial 3 → 3 + 1. As described above, this image specificity of WM neurons emerges during the fixation task, when most WM layer cells are quickly recruited into one of four groups, each containing cells responsive to a single image among the four used for the task. The remaining neurons that do not fall into any of these four dominant classes are found to be either always silent (class C_WM_, Figure 7) or to respond to several images (class F_WM_, Figure 7). As is the case for live subjects, we found that the relative number of cells responding to each image varies from one network to the other. This variability is caused by random factors such as the network's initial connectivity and the particular order in which images were presented to the immature model.

**Figure 8:**
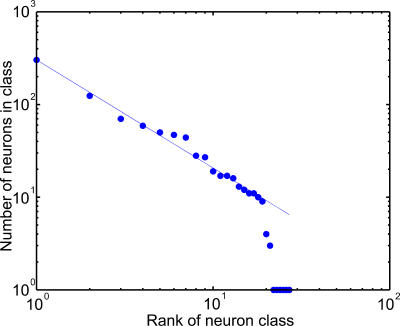
Distribution of WM Cells according to Their Firing Patterns Neurons were first grouped into classes according to their firing patterns during DMS and DPA trials (See Figure 7 for a list of the main classes). Classes were then ranked according to the number of neurons they contain, class 1 containing the largest number of neurons, class 2 the second largest number, and so on. Then, the number of cells in each class was plotted against its rank on a log–log plot. The straight fitted on the data obeys the power law: number of cells = 303 × (neuron class rank)^−1.66^.

Figure 6 also illustrates the high dependence of layer WM neuron activity on the trial period and trial type. Although the main role of the working memory layer in the network's dynamics is to sustain image-specific activity (i.e., to maintain a stable neural representation of images), Figure 6 shows that it can harbor several different representations for each image. Indeed, as stated in its legend, Figure 6 illustrates the presence of three representations of image 3: the evoked representation which is generated during the cue period of DMS trial 3 → 3 + 1, the sustained representation present during the subsequent delay of the trial, and the recalled representation which is generated during subdelay d3 of DPA trial 4 → 3 + 1. Similar sets of representations also exist for the other three images used in the task. This diversity finds its source in the firing of individual WM cells, which is not only tuned to images, but also depends on the task performed and the period of the trial.

Figure 7 presents a classification of WM neuron activity. Each class shown was obtained by grouping together cells responding to different images but presenting similar firing patterns. Although the relative size of the classes varied somewhat, we found the essential characteristics of this classification to be robust from one run to the other.

Type A_WM_ cells are the most common, firing whenever the cell's preferred image is perceived, kept in memory, or recalled. Such cells therefore participate in all three image representations mentioned earlier. Cells in the other classes exhibit similar firing patterns though they lack certain of its components. For instance, type D_WM_ units respond to the presentation of images, but they are unable to fire when the network recalls this image or sustains its representation. They therefore only participate in the evoked representation of images. By contrast, type H_WM_ cells only fire when images are retrieved during DPA trials. They consequently only contribute to the representation of recalled images. Type B_WM_ cells, which do not fire during the cue period, participate in the sustained and recalled representations of images, but not the evoked one. A similar analysis can be extended to the remaining classes of Figure 7. This classification, as well as the functional importance of neurons with response types A_WM_, B_Wm_, and D_WM_, are further supported by similar experimental [16] and theoretical results [17] obtained from monkeys and humans performing a delayed-response oculomotor task where locations, instead of images, must be remembered by the subject.

The diversity in firing patterns present in layer WM is a direct consequence of the structure of the network. Indeed, as shown on Figure 2, layer WM cells are the target of bottom-up connections from visual representation layer VR and top-down projections from the planning layer P; they are also part of the dense array of horizontal excitatory and inhibitory connections present within layer WM itself. Each cell is therefore subject to visual, recall, and reentrant signals, respectively, the relative magnitudes of which differ from one cell to the next as a result of the random nature of the network's connectivity. With these signals interacting in a nonlinear fashion, the possible firing patterns are quite numerous. Indeed, in the particular run used to produce the data presented in Figure 7, WM neurons could be distributed in no less than 27 different classes of neural activity. Figure 8 shows that their size distribution follows a power law (see the legend of Figure 8 and Discussion for details).

#### Layer P cells.

The activity of mature P cells holds more information than that of neurons in layers VR and WM: it depends not only on the images submitted to the network during the current trial, but also on the processing applied to these images (i.e., sustaining the memory of the sample image or recalling the image associated with it).

**Figure 9:**
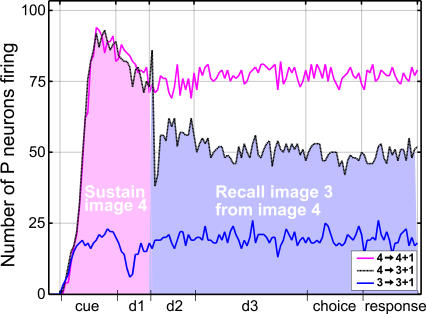
Evolution of Planning Activity during DMS and DPA Trials Each line represents the number of layer P neurons that fire at every instant during DMS and DPA trials. The pink and blue curves correspond to DMS trials where images 4 and 3 are used as sample, respectively. All P cells active during these trials are primed by the sustain task unit, and code for the project of sustaining the representations of sample images 4 and 3, respectively, that are harbored in layer WM. The black curve represents the number of cells firing during a DPA trial where image 4 is the sample and image 3 is the target. The light pink area denotes cells firing to sustain the representation of sample image 4. This set of cells has a large overlap (fluctuating between 75%–90%) with the cell population that was firing during the same period of the 4 → 4+1 DMS trial (pink curve). Such variability is a direct consequence of the randomness inherent to the network's dynamics. At the beginning of subdelay d2, when the network is instructed to perform the DPA task, activity in the task layer switches from the sustain to the recall unit. This abrupt modification in P cell priming creates a sudden reorganization of the cellular activity present in the layer: all previously firing layer P neurons are now primed into a quiet state by the silent sustain unit. Simultaneously, all cells primed by the now active recall task unit are free to fire. Those that do fire form the representation of the project to recall image 3 (light blue area).

This task-image duality in the cells' firing is produced by the combined effects of the priming connections projected onto layer P by the sustain and recall units of the task layer, and the image-specific signal sent from layer WM. Since two processing actions are possible (sustain and recall), and a total of four images are used for the task, P cells part themselves in eight different groups according to their function in the network's dynamics (e.g., sustaining sample image 1, recalling target image 1, etc.). The first four groups each contain cells primed by the sustain unit, stabilizing the representation of one among the four images used in the MDR task. Figure 9 illustrates the evolution over time of the activity of cells belonging to the group that codes for the project of sustaining image 4 during DMS (4 → 4 + 1) and DPA (4 → 3 + 1) trials. Cells in the remaining four groups are primed by the recall unit. They each generate in layer WM the representation of a different target image, and are therefore only active during DPA trials. Figure 9 illustrates the onset of activity of cells in one such group, which codes for the project to recall image 3 (trial 4 → 3 + 1).

**Figure 10:**
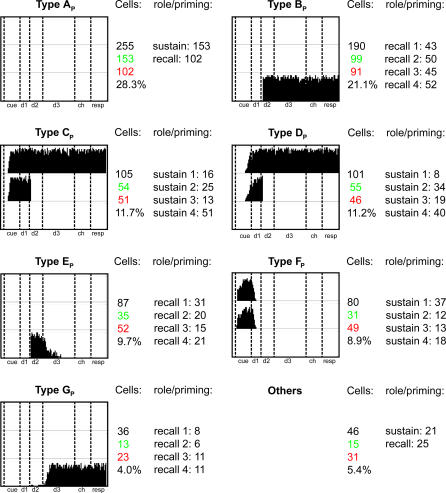
List of the Neural Responses of Layer P Units Layer P cells have firing patterns which code for either memorizing or recalling an image. Each box contains three raster plots (top, center, and bottom), which represent the response of a single cell to a DMS trial where the cell's preferred image has to be sustained (top), a DPA trial where the cell's preferred image has to be sustained (center), and a DPA trial where the cell's preferred image has to be recalled (bottom). Cells: For each class are listed the total number of cells in the class (black), the number of excitatory/inhibitory cells in the class (green/red), and the percentage of the total number of P neurons this class represents. Role/priming: For each class, the table specifies whether neurons are involved in sustaining sample images, or recalling the targets. It also specifies the image specificity of the cells. For type A_P_ cells and “Others,” we only specify the number of cells primed by each unit.

Figure 10 presents a classification of neural activity of layer P units where we have put together cells with similar firing patterns. We found that cells group themselves according to the priming connection they receive: type C_P_, D_P_, and F_P_ are all primed by the sustain task unit, while type B_P_, E_P_, and G_P_ cells are primed by the recall unit. Type C_P_ and B_P_ cells exhibit the most tonic discharge, being active whenever their priming unit is also firing (compare with firing patterns of recall and sustain units in Figure 3). Type D_P_, E_P_, F_P_, and G_P_ cells have similar, though incomplete, activity patterns, similar to what was observed in layer WM. Finally, type A_P_ cells exhibit very low activity or none whatsoever during DMS and DPA trials. It should be noted that, layer P being a higher processing area which does not receive any visual signal, P cell activity is less diverse than that found in layer WM.

### Evolution of Neural Activity during DPA Training

After successful completion of DMS training, the network is able to recognize images, sustain their representation during a delay, and pick them during the choice period. To pass the DPA task, the network must in addition be able to recall the image associated with the sample (i.e., to create a recalled representation of the target image from a learned association).

**Figure 11:**
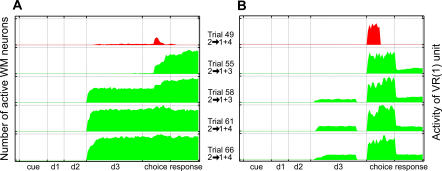
Recall of Image 1: Emergence of Prospective Activity in Layers WM and VR during DPA Training In the particular run used to gather the data plotted in the figure, DMS training was completed at trial 48, and DPA training started at trial 49. For clarity, we only present on this figure the trials where images 2 and 1 are the sample and the target, respectively (activity of cell populations during the trials in between, which feature other images, are not represented). Trials failed by the network are represented in red. Succeeded trials are displayed in green. (A) The areas represent the number of WM cells tuned to image 1 that fire at every instant of DPA trials where this image is the target (i.e., where image 1 has to be recalled from association). The figure shows the evolution of neural activity coding for the recalled representation of image 1. It first appears during the choice period of trial 55 as the network is presented with this image. At the next “2 → 1” trial (trial 58), this firing has become prospective, appearing at the start of subdelay d3 before image 1 has been presented to the network. The number of active cells varies between 0 and 120. (B) The area represents the activity of the excitatory cell VR(1). As discussed above, VR cells act in the network as visual extension to the content of working memory layer WM. VR(1) activity therefore virtually mirrors that emerging in layer WM during training, as can be seen by comparing the buildup of prospective activity in (A) and (B).

As described earlier, the network learns to associate images in pairs by a process of trial and error. After the successful completion of DMS training, layers VR and WM act as a “winner-takes-all” network during the choice period: when presented with the target and distractor images, the network is constrained to select one or the other. Which one the model chooses depends on the activity currently harbored in working memory: whatever image is currently memorized in layer WM will be chosen by the model (see Figure 4 and [13] for details). If little or no activity is present in the working memory layer during the choice period, as is the case during the first few trials of DPA training, the network will just pick an image at random between the target and distractor. This is illustrated by trial 49 of Figure 11A and 11B, where the network picks image 4 (i.e., the distractor) instead of target image 1.

The associations between images are coded in the network by the connections projected by layer P neurons onto layer WM cells. Taking the example of associating target image 1 with sample image 2 (illustrated in Figure 11), the network needs to strengthen connections that P cells coding for the plan of recalling image 1 project onto layer WM cells tuned to image 1. It is the resulting set of connections that stores the particular association in the network's circuitry.

Reward signal and reinforcement algorithm are crucial to selectively strengthening these connections, therefore securing the correct associations and discarding incorrect ones. Choosing the distractor image triggers the release of negative reward, which resets, through the reinforcement-learning algorithm, all connections projected by active P cells onto firing WM neurons (trial 49; Figure 11). Choosing instead the target reinforces the connections that led to the correct choice (i.e., the connections that P neurons coding for the plan of recalling a target image project onto WM cells selective for that image). The synaptic strengths of the connections will keep increasing at each successful trial.

This evolution in the network connectivity leads to modifications in the activity of layer WM neurons, as a new recall component emerges to complement their previous firing pattern. Further, Figure 11A illustrates at the population level that the onset time of this newly acquired activity changes during the course of learning. Indeed, at the first successful trial, WM cells start to fire during the second part of the choice period and throughout the response period (see trial 55, Figure 11A). However, at the next “2 → 1” trial (see trial 58, Figure 11A), this new activity has become prospective: it appears already at the beginning of subdelay d3, therefore before the presentation of the target image. The instant when target recall takes place therefore shifts from the choice period to the beginning of subdelay d3 in just two trials.

**Figure 12:**
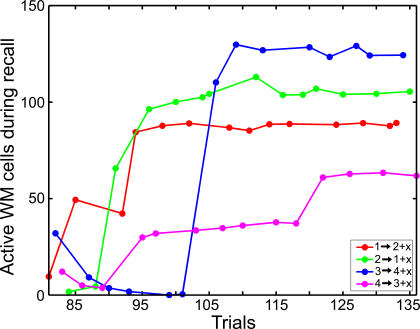
Evolution of the Size of Prospective Activity in Layer WM during DPA Training Each curve illustrates the evolution during DPA training of the average number of WM cells firing during subdelay d3, and tuned to a particular image. In the run used to produce the data plotted on the figure, DPA training started at trial 81. The graph includes both succeeded and failed trials: the number of active cells increases when the network receives positive reward, and it decreases when negative reward is dispensed to it. Each curve roughly follows a sigmoid function, reaching a plateau in a limited number of successful trials.

Figure 11A also illustrates that the number of cells which participate in the prospective recall of the target image increases during DPA learning. This is confirmed by Figure 12, which presents the average number of cells firing during subdelay d3 as a function of trials. The figure shows that this number varies gradually, following a sigmoid curve. The actual pace of this transition in neural activity is largely dependent on the value chosen for the parameters of the reinforcement-learning algorithm. For instance, smaller values of these parameters would lead to a slower modification of the network's connectivity, and consequently, of WM cell activity.

Figure 11B shows that the emergence of recall activity in layer VR mirrors closely that exhibited by WM neurons: it appears as soon as the correct target is first chosen, and then becomes prospective at the next “2 → 1” trial. This is not surprising, as VR cells act as visual components which complement the content of working memory layer WM. This prospective activity in layer VR during target recall is therefore entirely controlled by the higher areas WM and P and the connections they exchange: layer VR cells do not play any active role in the process of target recall, nor do they contribute to the neural coding of associations between images.

### Comparison with Experimental Data

The network dynamics during the task compares well with behavioral and electrophysiological data gathered on the monkey performing similar tasks.

Just like the monkey, the network solves the MDR task in a prospective manner: they both take advantage of the task-specific signal presented during the delay to predict and retrieve the upcoming target image before it is actually shown. This prospective strategy for passing the task was demonstrated for the animal by a detailed analysis of error patterns, reaction time, and electrophysiological data [12]. In the case of the network, it can be readily verified that the representation of target image 1 is created well before the end of the delay for the DPA trial (Figure 4, DPA d3-a).

At the neural level, it can be seen that the firing pattern of VR layer neurons (Figure 5A) reproduces the pair-suppression and pair-recall effects observed in the IT cortex (Figure 5B) of monkeys performing the pair-association with color switch task [11], a task virtually identical to MDR. Indeed, neuron VR(2) exhibits both a suppression of its firing during the delay when the representation of sample image 2 is eliminated (Figure 5A**,** 2 → 1 + 3), and an activity enhancement starting during the delay when the network recalls target image 2 (Figure 5A, 1 → 2 + 3). Further, as shown by the overall dynamics of the network (see Figure 4), the model also agrees with the interpretation that the pair-suppression and pair-recall effects are evidence at the cellular level that, during DPA trials, activity in the IT cortex specific to the sample image is replaced during recall by a new activity specific to the target [11].

**Figure 13:**
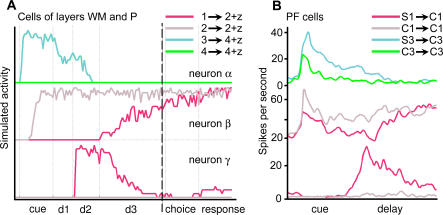
Comparison of the Activity of Simulated Units of Layers WM and P with that of Cortical Neurons Comparison between simulated (A) and experimental (B) units is restricted to the cue and delay periods (left of the dashed line on theoretical data), although the model makes predictions for the choice and response epochs as well. Notation: x → y + z = trial with sample x, target y, and distractor z. Other notations are as in Figures 1 and 2. (A) Average firing patterns of cells of layer WM (neurons α and β) and layer P (neuron γ). For clarity, each graph represents the response of cells for only two trials (their firing for the other two trials being at background level). Task parameters and notations are as in Figures 1 and 2. Task parameters: cue, 0.5 s; d1, 0.3 s; d2, 0.4 s; d3, 1 s; choice, 0.5 s; response, 0.5 s. (B) Recordings performed in monkey dorsolateral PF cortex (modified from Figure 5 of [12]). Activity was recorded in animals trained with six stimuli (Si and Ci [i = 1, 2, 3]) to perform a task of randomly mixed DMS and DPA trials. Images Si are only used as samples for DPA (and associated with images Ci), while stimuli Ci serve as targets in DPA trials and samples/targets in DMS trials. Here, for clarity, we reproduce only the response of each cell for two different trials (The response of cells for the other trials presented in Figure 5 of [12] is much smaller and mainly confined to the cue period). Task parameters: cue, 0.5 s; delay, 1 s.

We now compare the activity of neurons in the WM and P layers (Figure 13A) with the firing of cells measured in the dorsolateral cortex of monkeys (Figure 13B) performing an analog, though slightly different, pair-association task [12]. In this case, there is no task-specific signal during the delay to specify the trial type. Instead, the monkey can deduce from the sample images used whether the current trial is of type DMS or DPA (see Figure 13B legend). Though not a perfect match, the simulated units do reproduce important features of the stimulus-specific firing of the PF cells.

The cell illustrated at the top of Figure 13B has a firing that is both specific to sample image S3 and mainly localized to the cue and early part of the delay. This firing pattern resembles the activity of WM neurons contained in classes G_WM_ (e.g., neuron α, Figure 13A) and D_WM_ of Figure 7, or that of P neurons belonging to class F_P_ of Figure 10. This interpretation suggests that the measured dorsolateral cell takes part in the evoked representation of image S3. Alternatively, it could also indicate that this cell takes part in the plan of using image S3 as target.

The cell illustrated in the middle of Figure 13B exhibits a firing correlated with image S1, whether this image is presented to the animal, memorized, or recalled during the delay. This activity is analog to that of WM layer neurons belonging to class A_WM_ of Figure 7 (e.g., neuron β, Figure 13A). According to the network, the measured dorsolateral cell would then take part in the evoked, sustained, and recalled representations of image S1.

The last cell (Figure 13B, bottom cell) displays a richer, task-related firing. Contrary to the previous cell, which fired whenever image C1 was used as target, this neuron is only active during trials where image S1 has to be recalled: it is silent during DMS trials where image C1 is the sample and only needs to be remembered during the delay. These activity characteristics are shared with neurons in classes H_WM_ (Figure 7). Indeed, WM cells grouped in class H_WM_ only take part in the recalled representation of images. However, more interestingly, the activity of this dorsolateral cell also matches the firing of *P* neurons contained in class E_P_ (Figure 10). These cells are part of the neural assemblies in layer P that code for the project of recalling the target image (neuron γ in Figure 13A belongs to this class). According to this interpretation, the measured cell would therefore take part in the plan of recalling image C1 by the animal.

### Robustness of the Dynamics

We tested the robustness of the model's dynamics at the neural and network structure levels by modifying parameters such as the membrane time constants, the parameter defining the overall scale of the cells' threshold, the misfire probability of cells, or the number of connections present within or between layers. In all cases we found that network performance varied either little, or very gradually, as a function of these changes.

**Figure 14:**
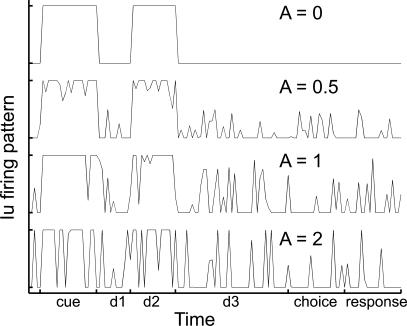
Perturbation to the Firing Pattern of Control Unit I_u_ To test the robustness of the network's dynamics, the firing patterns of control units were perturbed by adding to them white noise of varying strength A. The top curve (*A* = 0) shows the unperturbed firing pattern for gating I_u_. It is binary, being either equal to 0 (i.e., gating I_u_ fully closed) or 1 (I_u_ fully open). The next three curves are examples of activities perturbed with noises of amplitude *A* = 0.5, 1, and 2. There, activities are no longer binary as they can take any value between 0 and 1. Such intermediate values correspond to gating I_u_ being partially open (i.e., transmitting only a portion of the information traveling from layer WM to layer P). A similar analysis extends to disturbing the other control units of the model.

**Figure 15:**
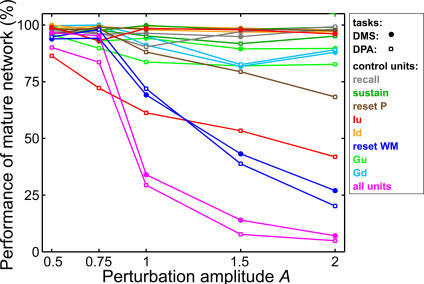
Effect on Network Task Performance of Perturbing the Control Units' Firing Patterns Each curve represents the success rate of the mature network for a given task when the firing pattern of one or all control units are perturbed by noise of amplitude *A* (each performance number has been obtained by compiling network success over at least 5 runs). Data corresponding to each curve are specified both by the marker used (dot for the DMS task, empty square for DPA) and the color of the curve that indicates which control unit has been disturbed. For most units and tasks, network performance varied little with increasing perturbation amplitude. In the other cases however, we found a marked sigmoid-type decline in performance.

We also studied at the systemic level the effect on the network of perturbing the control units' firing patterns. Indeed, out of simplicity, the results described above where obtained using simple binary activities for all control units (see Figure 3). To test whether binary firing patterns where essential to proper task performance, we perturbed them with white noise of increasing amplitude *A* while monitoring network performance separately for the DMS and DPA tasks (see Figure 14 and its legend). The process of evaluating the effect of these perturbations on the dynamics unfolded as follows. Each run took place as usual during the training phase, with the network using the binary firing patterns of all control units shown on Figure 3. Once training for all tasks was successfully completed, we then added white noise of amplitude *A* to the firing pattern of either one or all control units. Figure 14 shows that the resulting perturbed firing pattern is now no longer binary and can take any value between 0 and 1. As the amplitude *A* of the noise increases, the unit's activity looks less and less like the original, unperturbed binary firing pattern. Network performance is then monitored for each value of *A* and the percentage of success for the DMS and DPA tasks recorded separately (see Figure 15).

Simulations showed that perturbing a single or all control units produces little or no effect on the network's dynamics as long as *A* is roughly smaller or equal to about 0.5, even though the effect on the control units' firing pattern is already quite noticeable (see Figure 14). This therefore reveals that the mature network does not require binary firing patterns for its control units.

Increasing the value of *A* further brings the system in a regime where the perturbation is equal in size to the signal itself (Figure 14, *A* = 1 and 2), to the point where noise actually alters the very structure of the activity patterns. In the example portrayed in Figure 14 (*A* = 2), gating I_u_ closes during the cue and d2 periods when it should remain open, thereby simulating a brief misfire of the unit. The added noise also opens the gating during the d1, choice, and response periods (i.e., simulating large spontaneous activity for the unit). Such effects are also affecting the functions of gatings I_d_, G_u_, and G_d_. Similarly, perturbations on the firing pattern of the sustain and recall units can briefly activate these two units at the same time, simultaneously triggering cells in layer P that code for sustaining the sample image and others that implement the recall of the image associated with the sample. Finally, perturbation of the reset WM and reset P units can either lead to the repetitive suppression of ongoing activity in layers WM and P at any moment of the trial, or missing the proper initialization of layers WM and P.

Figure 15 presents the effect of these perturbations on network performance. Their impact, very pronounced in the case of three control units, reflects the respective role played by the latter in the network. For instance, perturbing the activity of reset WM unit deteriorates the network's performance for both the DMS and DPA tasks. Indeed, the repeated activation of this unit systematically suppresses from layer WM an activity that is crucial to all the network's computations. By contrast, adding noise to the activity of gating I_u_ or the reset P unit only affects DPA task performance, as layer P is not essential to passing the DMS task. For some units (e.g., sustain and recall), no significant loss of performance was observed at all. For the remaining units, the perturbations had only a limited impact on network performance, except when they were applied to all units simultaneously. We also found that even for noise amplitude *A* = 1, the system could still function to an acceptable level when the firing pattern of only a single unit was modified.

This resistance to local perturbations stems from the structure and dynamics of the network itself: the system is cooperative by construction with distinct units interacting to process the necessary neural computations. If one unit misfires, thereby failing to produce the activity required for the next step of the task, the results of previous neural computations will most likely still be present in the network until the perturbed unit does decide to fire. The reset of layer WM during DPA trials is a good example: even if unit reset WM eliminates repeatedly during subdelay d3 the activity in layer WM coding for the recalled target image, planning activity coding for the recall of this target is still safely stored in layer P and ready to be used during the choice period. In addition, the lateral inhibition present in the network is sufficient to minimize, or even control, the effect of any parasitic activity created by the spontaneous firing of control units. An example of such activity is the firing during DMS trials of layer WM and P cells tuned to the recall of the image associated with the sample. It arises when perturbation are applied to the firing pattern of the recall unit, and it can interfere with the behaviorally correct activity harbored in layers WM and P coding for the sample image. However, as shown on Figure 15 (recall unit and DMS task), the network's lateral inhibition manages to keep this parasitic activity from affecting DMS performance.

The ability of layers WM and P to sustain activity gives considerable leeway to the system: to pass the DMS and DPA tasks, the system must accomplish a series of neural computations in a precise order, and in a manner synchronized with the periods of the task. However, because of the ability of layers WM and P to sustain activity for up to several seconds, the precise instant when these computations take place, or even if they are repeated several times, have little influence on the network's performance. This explains the limited loss of performance when damaging most gating units.

We found the system's dynamics to be much more sensitive to perturbations during the training phase (not shown). We observed in fact that the earlier perturbations are taking place, the more severe their effects are on the dynamics.

This result is a direct consequence of the way in which circuitry emerges in the network. Indeed, as described above (see Learning Phase), network organization starts with the construction during the fixation task of neural clusters in layers WM and P, whose activity represents images and plans, respectively. As long as this crucial first phase is not totally completed, neural circuitry in layers WM and P is extremely labile: immature clusters can easily fuse together, depriving the network of the ability to treat different images or plans as distinct objects, and therefore ruining any chance of successfully completing the training phase. Such cluster collapse is certain to take place if information travels back and forth between pairs of layers (e.g., layers VR and WM, or WM and P). To keep this from happening, it is therefore essential that during this early part of training, gatings G_u_ and G_d_ (and I_u_ and I_d_) are never open at the same time [13].

This provision is no longer satisfied when large perturbations are added to the firing patterns of all control units, since they will certainly generate frequent simultaneous opening of gatings G_u_ and G_d_, or I_u_ and I_d_. This will in turn result in the collapse of clusters in layers WM and P, and the complete breakdown of network performance for all tasks. In contrast, starting to apply these perturbations later during training (i.e., when cluster integrity is already higher) will have a smaller effect on network circuitry and task performance.

## Discussion

### Comparison with Other Models

To our knowledge, two models, each using attractor neural network dynamics, have been proposed so far to study the neural mechanisms implementing memory recall in the framework of the DPA and DMS tasks [18,19]. The first was proposed by Morita and Suemitsu [18], and the second by Brunel and colleagues [19,20]. Both approaches reproduce electrophysiological data gathered on monkey IT cortex during DMS and DPA task performances, and make testable predictions.

These two models have in common with the present work the use of re-entrant connections to produce stable, image-specific activity representing the observed sample image and the expected target image. However, in our case, neurons are arranged in two-dimensional layers and the excitatory connections which link them are exclusively short range. Re-entrant neural circuits therefore organize themselves locally in the layer instead of spanning the whole network, as is the case in the above two models.

In all three models, the retrieval of the target image takes place as the system leaves the attractor corresponding to the activity coding for the sample image, and moves toward that coding for its paired-associated. However, the different mechanisms through which this is achieved, and how associations between images are built, differ sharply. While in the models described above [18,20] direct connections between cells coding for the sample and target images induce the transition, in the present one it is the vertical connections that are exchanged between the separate working memory and planning layers that code and implement the memory retrieval. Further, the recall process is monitored and guided by control units (reset WM, I_u_, and I_d_), instead of depending on microscopic parameters of the model and on its level of spontaneous activity. This completely different dynamics allows more behavioral flexibility, as firing patterns can be modified to suit new task parameters (e.g., longer or shorter delays).

Also, in the attractor neural network models above, associations are constructed through a slow process (either through Hebbian or more complex guided forms of learning) during the choice period of trials where neural activities coding for the sample and target images coexist. In that framework, the notion of reward, which is an intrinsic part of the protocol for the DMS and DPA tasks with live subjects, is absent from the dynamics of the learning or mature network. The network in the present work adopts the complementary view: reward is essential to DPA training, and is used by the network as a guide to learn the correct associations and discard incorrect ones. Indeed, during DPA trials, the network picks one of the two images presented to it during the choice period. This image is then the candidate that the network tentatively suggests to form a pair with the sample image presented before the delay. The reward signal dispensed to the network by the “experimenter” is then used by the model to figure out whether this choice is correct or incorrect.

It is interesting to compare the characteristics of these models with the known mechanisms for the retrieval of memories in primates that were studied using MDR-type tasks [2,11,12]. According to recent findings, two parallel pathways for factual (or semantic) memory have been identified in the monkey [21]: one, for automatic retrieval, only involves the medial and temporal lobes [22], while the other, for active retrieval, runs from the frontal cortex [23]. We suggest that the attractor neural network models [18–20], which were designed to model IT circuitry and function without the intervention of higher cognitive areas such as the PF cortex, implement essential features of the mechanism for automatic retrieval. On the other hand, we propose that the present network, which models the prefrontal and temporal cortices, would be closer to the mechanism for active retrieval system. This would put forward the assumption that, in delayed-response tasks, associations might be partially coded in the PF cortex instead of solely in the IT cortex as is often assumed in the literature. The two theoretical approaches described above therefore seem to us complementary and should be able to coexist in a unified framework.

### Dynamic Properties of Network Connectivity

Behind the complex behavior exhibited by the model as it passes the DMS and DPA tasks, there is an interesting dynamic that takes place in the network both at the cellular and circuit levels. Indeed, classification of WM neurons according to their firing patterns, revealed that the distribution of cells in these classes follows a power law (see Figure 8 and legend for details). Power law distributions are usually the signature of intricate dynamics and of a certain degree of scale invariance in the system (see, for instance, [24] for a review and references therein). In the present case, this distribution suggests that despite the short-range horizontal connectivity present within layer WM, the firing pattern of each WM cell might organize itself in a global manner (i.e., by taking into account the activity of all other cells in the layer). This process could take place in a cooperative manner as suggested by the presence of sigmoid curves in the dynamics of the network either when circuits are created (Figure 12) or damaged (Figure 15). The wide array of possible neural activities present in layer WM might then prove essential to the stability of clusters: the network could probably not function correctly only with cells of, for instance, type A_WM_ (see Figure 7). An organization of layer WM of this type would prove very unstable. Although scale invariance has already been observed in several aspects of brain dynamics and structure [24], such effects have to our knowledge yet to be reported in neural network models of cognitive functions.

### Appraisal of the Model Assumptions

Though simple compared to the central nervous system, the present model captures key biological features of several brain areas thought to take part in the neural processing necessary for the DMS and DPA tasks. We will examine each of them below.

The model manages to be trained for both the DMS and DPA tasks using two biologically realistic synaptic modification algorithms. The first is the Hebbian [14] learning mechanism, which is usually interpreted as being implemented by the phenomenon of long-term potentiation and depression [25] in NMDA synapses. The other, reinforcement learning, is a generalization of the Hebbian synaptic modification algorithm, which makes use of the reward signal dispensed to the subjects and the presence of neurotransmitter pathways linked to reward (see [26] for a review). In order to account for the high connectivity present in cortex, synapses in the model can be interpreted as representing either single cortical synapses or an ensemble of synapses.

Moreover, the layers of the network implement functions typical of known brain areas or regions.

Layer VR is the point of convergence of both the bottom-up visual component coming from primary visual areas, and the top-down signal originating from working memory. As was shown when comparing simulated and experimental data, the activities of VR neurons fit those measured in the IT cortex during the cue and delay periods of both DMS and DPA trials [2,11]. The firings of VR cells during the choice and response periods also capture key features of electrophysiological data gathered in the IT cortex of primates [27] during the target selection process of DMS trials [13]. Altogether, these observations therefore suggest that layer *VR* simulates in the model networks present in the primate IT cortex.

Layer WM implements in the model several functions typically identified with PF circuitry: it is capable of sustaining activity coding for objects, whether they are perceived [16] and memorized [3,16] or recalled [12], and it has some control over the activity in higher visual areas [27,28]. This tentative identification of layer WM with circuits in the PF cortex is further comforted by the qualitative agreement between activities of WM neurons and that of dorsolateral cells.

Both these interpretations are further supported by the qualitative agreement between the connectivity linking the PF and IT cortices, and that exchanged by layers VR and WM: they are both dense, two-way, and also subject to strong reward-type innervation [29–31]. All these characteristics are absolutely crucial in the model to the emergence, during DMS training, of the target selection mechanism which spans layers VR and WM, and that is subsequently used when performing the DPA task.

The planning activity stored in layer P introduces a prospective component in the network's behavior. Planning results from the convergence of task-related signals and activity emanating from the image representation held in working memory. This characteristic firing pattern, which as shown above fits well observed data, brings support to the hypothesis of a memory retrieval mechanism relying on planning circuits harbored by the dorsolateral cortex. The assumption that the planning circuitry required for the DMS and DPA tasks is located in the PF cortex agrees with experimental studies, suggesting a role for the PF cortex in tasks involving high-level planning, such as the Tower of London task [32].

Units in layer T, which implement task-related higher processing, have a function similar to cells that were observed in monkey PF cortex and whose firings are specific to tasks [33] or abstract rules [6]. According to the present model, a minimum of two distinct task-related processings should be required for the MDR task: one would come into play when the subject retains the sample image, and the other would be active during the recall of the target associated with the sample.

The reset units of the model, which are dedicated to removing behaviorally irrelevant activity from the network, have a function close to the “inhibitory control” proposed by Fuster [34] and which neuropsychological and neurophysiological studies place in the orbitomedial PF cortex [34]. Such mechanisms form the necessary counterweight to the property that neural systems possess of sustaining the activity they harbor. They were studied both theoretically in the framework of attractor networks [35], and more recently experimentally, where they were linked to executive functions and planning [36].

Gating units G_u_, G_d_, I_u_, and I_d_ provide the model with the ability to control the flow of information it harbors. Several experimental studies seem to indicate that such mechanisms exist in the brain. One example is the electrophysiological study by Miller et al [28] on primates performing the so-called running-DMS task. This task differs from ordinary DMS by the successive presentation of a varying number of distractor images before the target is actually displayed. The subject is required to withhold its response until the target appears. Recordings showed that IT cells exhibited a firing highly modified by the images presented, while PF cells have a more stable response over the trial. These results indicate that activity does not circulate freely between the IT and PF cortices, and therefore suggest the existence of a mechanism that shields PF neurons from behaviorally unimportant visual information. Electrophysiological data gathered by Chelazzi et al [27] on the primate performing the DMS task also point toward a mechanism which manages the flow of information between the IT and PF cortices to allow the animal to pick the target image while ignoring the distractor (see [13]).

In addition to these observations, mechanisms to shield information from competing neural activity or to manage the flow of information along neural pathways have already been proposed in the PF cortex [37] and the basal ganglia [38,39], respectively.

Indeed, Sakai et al. [37], using an experimental setup which requires that subjects keep in mind information while performing a nonrelated distractor task, have showed that area 46 of the PF cortex seems able to implement a type of shielding mechanism allowing different information to be simultaneously held in the brain. This shielding mechanism is very similar to the influence gatings I_u_ and I_d_ have over layers WM and P, which, for instance, permit representations of both the sample image and the project to recall the sample's paired-associate image to coexist in the network during subdelay d2 of DPA trials (Figure 4, DPA d2).

Also, Graybiel and colleagues [38] proposed that a type of gating mechanism might be implemented by so-called projection neurons in the striatum. These neurons tend to exhibit a bursting firing pattern, switching between an “up” and a “down” state. Their connectivity also makes them susceptible to enhance or suppress movement through the basal ganglia pathways, which run from the neocortex, then through the striatum and other parts of the basal ganglia, and finally converge to frontal areas of cortex. The analogy between this circuitry and the gatings of the model is especially interesting as data also seems to indicate a role for basal ganglia at the level of planning and cognition [39].

We suggest that the regulation of the flow of information between the PF and IT cortices illustrated by the present model could be implemented either by separate loops which have been reported as linking the basal ganglia with the temporal association area TE via the thalamus [40] and the basal ganglia together with the PF cortex [41], or alternatively through yet undiscovered more direct circuits spanning the PF cortex, IT cortex, and the basal ganglia (see Figure 2).

### Toward a Representation of the Tasks Themselves

In order to pass the DMS and DPA tasks, live subjects must have an understanding of what they are required to do. In other words, they must hold in their mental space a neural activity that represents these tasks, or at the very least, which codes for the different operations required to perform them. Animals are able to create this neural task representation in a few training sessions, using the reward dispensed by the experimenter as only feedback on their actions from the exterior world.

The present model in contrast does not learn the DMS and DPA tasks: all the computational aspects of the tasks are already precoded in the firing patterns of the network's control units (see Figure 3). During the training phase, this abstract task representation is then applied to the images chosen for the task, as the network modifies its connectivity and becomes able to pass MDR trials with that particular set of images. However, despite these simplifications, the model is able to suggest some insights into the neural activities that implement behaviors.

One important question it addresses is how much the behavior of a live subject is sensitive to the details of the neural activity that codes for it, or, in other words, whether a given task has only one unique neural representation. The present model seems to suggest that it is not the case. Indeed, as we saw above, the network can function with an acceptable degree of success in spite of very extensive perturbations to the firing patterns of its control units. This clearly indicates that there is a large spectrum of firing patterns that the control units could adopt that would still lead to task success. This is partially due to the fact that, as mentioned earlier, after just a few trials, information sustains and protects itself in the network with reentrant excitatory connections and lateral inhibition. From then on, the exact timing of neural operations, or the number of times they are repeated, seems to be irrelevant as long as they occur in the right order and each within a certain time window. The firing patterns displayed in Figure 3 and used to produce the results presented here, were chosen arbitrarily because they were thought to offer optimal network performance. However, there appears to be a host of different firing patterns (i.e., neural representations for the task), which would provide equal or near equal success rates. This compliant character of the model stems directly from its modular organization and its ability to sustain and protect information, raising the possibility that the same is true in the brain of primates.

## Materials and Methods

### Neuron modeling.

The network is composed of leaky integrate-and-fire neurons represented by three dynamical variables [13]: a membrane potential (*mV*), a firing threshold (σ *V*), and the output *V* of the neuron which is a binary variable equal to 1 if the neuron is firing and 0 otherwise. Neurons are stochastic and fire with probability *p* = 0.9 any time their membrane potential is larger than their threshold (to simulate a slight misfire). White noise has also been added to the visual input fed into layer VR by lower visual areas Input. For simplicity, we have not included any firing refractory period or membrane potential reset after firing. The membrane potential, thresholds, and the output of all the neurons in the model are computed one-by-one for each time-step *dt* = 0.025 s. This is done in an order randomly drawn at each *dt*. We use a variable firing threshold σ *V* for all neurons, which is defined as proportional to the sum of all excitatory inputs afferent to the membrane of the neuron. As demonstrated elsewhere [13], this definition is mathematically equivalent to the scaling of synaptic coefficient observed by Turrigiano et al. [42], and it preserves the ability of cells to differentiate between their inputs during the learning phase when synaptic coefficients grow by several orders of magnitude.

Units in the network are interconnected into layers that are themselves connected together, for a grand total of more than 85,000 connections. Each of the neurons forming layers WM (containing 900 neurons) and P (containing 900 neurons) are interpreted as representing a single cell of the PF cortex. Units in layers VR (containing eight units) are meant to model whole arrays of IT cortex cells. Therefore, before comparing VR variables with experimental data, we need to reinterpret as firing probability or rate of activity the difference *mV −* σ *V* further modulated by the gatings afferent on the cell.

### Learning algorithms.

Each connection between two neurons in the network is represented by a synaptic strength *J* subject to the arbitrary constraint |*J*| ≤ 1: *J* is positive for excitatory synapses and negative for inhibitory ones. The strength of each synapse varies as a function of time, being modified at each time step by ordinary Hebbian [14] and reinforcement learning algorithms following equation *J* (*t* + *dt*) = *J*(*t*) + δ *J*. The quantity δ *J* depends on the nature of the synapse and on the type of connection in the model. For all horizontal and vertical upward connections, δ *J* is only equal to the Hebbian synaptic modification term δ *Jh*: δ *Jh* = η *V_pre_*(*t*) *V_post_*(*t*) for excitatory connections and for δ *Jh* = η (1 − *V_pre_*(*t*)) *V_post_*(*t*) for inhibitory ones, where *V_pre_*_(*post*)_ notes the output of the pre(post)–synaptic neuron and η ~ 10^−4^. For downward vertical connections, δ *J* = δ *Jh* + δ *Jr* where δ *Jr* is the contribution provided by reinforcement learning. We define δ *Jr* = ρ *V_pre_*(*t*) *V_post_*(*t*) where ρ ~ 10^−1^ and *R* represents the reward signal which is equal to 0 during the whole trial except during the response period where it is equal to 1(−1) if the network has given the correct (false) response during the choice period.

### Gating, reset, and task units.

Gating units G_u_, G_d_, I_u_, and I_d_ are implemented in the model by binary variables that take the value 1 when the corresponding gating is opened, and 0 when it is closed (Figure 3A–C). These variables modify the membrane potential and firing threshold of postsynaptic neurons, being multiplicand of the synaptic strength of gated afferent connections [13]. Reset units, which eliminate ongoing activity in layers P and WM, have been implemented in the model as instructions that reset to 0 the membrane potential and output of cells when these units are “active.” The priming effect of task units sustain and recall on P cells is implemented in the network as multiplicand on the output of P cells.

### Description of a typical run.

Each run starts by building the network anew as connections, neuron types (either excitatory or inhibitory) in layers P and WM, and the task unit priming each P neuron are all drawn using a random number generator. The model is then subjected to fixation, DMS, and then DPA trials, with reward dispensed according to the rule of the task. We choose as success criterion for a task the completion of a series of 20 correct trials in a row where the network “points” unequivocally to the target during the choice period. Excitatory units of layer VR, which are unequivocally stimulus specific, are used to “read” the network's response since we did not model a motor cortex in the network. Once DMS training is successfully completed, all connections modified during this learning (i.e., connections exchanged by layers VR and WM, those exchanged by sustain-primed P cells and WM neurons, and those harbored within layer WM) are then effectively kept “frozen” during the subsequent DPA training. This precaution, not unlike the overlearning procedure used with animals, ensures that DMS knowledge is not damaged during the DPA learning phase. The synapses of projections exchanged between WM neurons and recall-primed P cells, which have not yet been recruited by the dynamics, will now be modified to complete the circuitry necessary for DPA task performance. Complete runs are then repeated a large number of times (typically 100) to measure the probability of success for the different tasks.

## Supporting Information

Video S1 Executing the DMS Test (8.3 MB MOV)

Video S2 Executing a DPA Test (8.1 MB MOV)

## Abbreviations

DMS: delayed-matching to sample
DPA: delayed-pair association
IT: inferior temporal
MDR: mixed-delayed task
PF: prefrontal
